# The influence of enrofloxacin, florfenicol, ceftiofur and E. coli LPS interaction on T and B cells subset in chicks

**DOI:** 10.1007/s11259-015-9632-7

**Published:** 2015-02-10

**Authors:** Chrząstek Klaudia, Wieliczko Alina

**Affiliations:** Department of Epizootiology and Clinic of Bird and Exotic Animals, The Faculty of Veterinary Medicine, Wrocław University of Environmental and Life Sciences, Pl. Grunwaldzki 45, 50-366 Wrocław, Poland

**Keywords:** Enrofloxacin, Florfenicol, Ceftiofur, LPS, FCM, Chicken

## Abstract

This study aimed to investigate the influence of enrofloxacin, florfenicol, ceftiofur and *E. coli* LPS interaction on T and B subsets in thymus and spleen of newly-hatched chicks. A 126, 1-day-old chicks were administered enrofloxacin, florfenicol or ceftiofur in recommended doses according to the currently treatment schedule advises. *E .coli* LPS was given intravenously once at the dose of 200 μg kg^−1^ BW on the 2nd day of experiment (d. e.). On the 6th and the 14th d. e. thymus and spleens were subjected to flow cytometry investigation.

The most significant changes were demonstrated in spleen. The antibiotics administration decreased the percentage of B and T cells subset. Moreover, this suppressive effect was enhanced by *E. coli* LPS administration. On the 6th d. e. the percentage of CD3^+^TCRγδ^−^, CD3^+^TCRγδ^+^, CD4^+^CD8^−^, CD4^−^CD8^+^ decreased significantly after ceftiofur and LPS treatment. A lower percentage of CD3^+^TCRγδ^−^, CD4^−^CD8^+^ and CD3^+^TCRγδ^+^ was observed in enrofloxacine and LPS treated group. The decrease percentage of CD3^+^TCRγδ^+^cells and Bu-1^+^ was found after florfenicol and LPS treatment. On the 14th d. e. a decreased percentage of CD4^+^CD8^−^ and increased percentage of CD4^−^CD8^+^ cells was shown in ceftiofur or enrofloxacine and LPS treated groups. In addition decreased percentage of CD3^+^TCRγδ^+^ was found in all antibiotic and LPS treated groups.

In this study, it was shown that enrofloxacine, florfenicol, ceftiofur treatment may change the proportions among lymphocytes subset and might have an impact on the immune response to bacterial endotoxins in chicks.

## Introduction

The immune response in birds during the first week of life depends generally on normal function of main lymphoid organs where differentiation and development of lymphocytes take places. Many factors have a direct or indirect influence on immune response in newly-hatched chicks. The antibiotic therapy applied in the first week of chicks’ life may serve as one of the factors affecting immune response. The enrofloxacin, florfenicol and ceftiofur are broad-spectrum antibiotics commonly used against many diseases in domestic animals, including poultry (Booker et al. 1997; Burton et al. 1996; El-Banna 1998; Khalifeh et al. 2009). So far, it was shown that enrofloxacine and florfenicol despite reducing the humoral immune response had beneficial effects on the cell-mediated immune response in chicken (Hassanin et al. 2014; Khalifeh et al. 2009; Tokarzewski 2002). However, little is known about antibiotics influence on inflammatory response in chicks. Lipopolysaccharides (LPS), cell wall components of gram-negative bacteria, can induce an acute phase response which is a critical response to the infection. Cytokines (such as IL-1, IL-6) are released during this response, which lead to an acute proinflammatory reaction (Aldred and Schreiber 1993; Rath et al. 2000). Intravenously administered LPS in chickens causes pulmonary hypertension and decreases total peripheral blood leukocyte concentrations at 1 h post injection (Wideman et al. 2001, 2004; Wang et al. 2003). Based on the flow cytometric analysis of the proportions among T and B lymphocytes within the PBMC suspension, it was shown that the drop in lymphocyte levels observed on the 1, 3, and 6 h post LPS injection was due to a proportionately greater drop in T cells than in B cells (Bowen et al. 2009). Visually, the birds seemed to overcome the acute phase response during the first 24 h however the metabolic changes (e.g., decrease level of bone calcium concentration and relative weight of the tibia, worst feed consumption, decrease of BW, increase of relative liver weight) still existed up to 72 h after LPS injection (Mireles et al. 2005).

On the other hand, it was also shown that some of the antimicrobial peptides have a LPS neutralizing activity. Rifkind and Palmer (1996) were the first to report that three polypeptide antibiotics, namely polymyxin-B sulfate, colistin sulfate, and tyrocidine hydrochloride, have a capacity to neutralize endotoxins. Recently, it was shown that LPS neutralization by polymyxin B causes an immediate decrease in NF-kB binding activity in already activated human PBMCs that have produced cytokines (Tsuzuki et al. 2001). To date, we have no data regarding LPS neutralization by antibiotics in chicken. We hypothesized that it may be possible that also in chicken cells similar mechanism might occurs. This study aimed to investigate the influence of enrofloxacin, florfenicol, ceftiofur and *E.coli* LPS interaction on T and B cells subsets in thymus and spleen of newly hatched chicks.

## Material and methods

### Animals

A total of 126 of 1-day-of-age healthy Hubbard Flex breeder males’ chicks were obtained from a commercial hatchery and were raised in cages. The 56 chicks from the first experiment were allocated randomly to 4 cages of 14 chicks each whereas 70 chicks from the second experiment were allocated randomly to 5 cages of 14 chicks each, on the first day of experiment (1-day-of-age chicks). The chicks were not vaccinated. The housing area was scrubbed and steam-cleaned before the birds’ arrival. Lighting and ventilation were identical for all the chickens. All birds were kept in laboratory conditions. Water and commercial feed were available ad libitum. They were monitored daily, and no clinical signs of disease were observed during the time of the experiment. The experiment was conducted with the consent of Local Ethical Committee for Animal Experiments.

### Experimental design

This experiment consisted of two parts. The chickens from the first hatch (*n* = 56) were allocated into the experimental groups of 14 animals each: E, F, C and the control group. The group E was given enrofloxacin orally (Enrofloxan®, Biofaktor, Skierniewice, Poland), according to the currently advised 5-day treatment schedule. Each day, the birds received a single dose of 10 mg/kg body weight enrofloxacin into the crop. Group F was treated orally, into the crop with a single dose (30 mg/kg bw) of florfenicol (Floron®, KRKA, Novo Mesto, Slovenia) applied to the crop each day during the 5-day treatment. The oral treatment was administered with the use of a feeding tube and a syringe in both groups. The group C received in first day of experiment single subcutaneous doses of 2.0 mg ceftiofur sodium (Cefur®, ScanVet, Skiereszewo – Gdańsk, Poland) per kg body weight, the recommended therapeutic dose according to the manufacturer’s instructions. The control groups (*n* = 14) received only equal volume of drinking water into the crop, as in the whole experiment, the water was an vehicle for antibiotic solution.

The chickens from the second hatch (*n* = 70) were allocated into the five experimental groups of 14 animals each: groups E+LPS, F+LPS, C+LPS, LPS and negative control (K-). Groups: E+LPS (*n* = 14) received enrofloxacine and *E. coli* LPS, F+LPS (n = 14) received florfenicol and *E. coli* LPS, C+LPS (*n* = 14) received ceftiofur and *E. coli* LPS. The antibiotics were given as described above. *E. coli* LPS (O127:B8, Sigma, LOT 048 K4001, 600,000 u/mg) was dissolved at 1 mg/mL in PBS and then reconstituted *in aqua pro injection* to the working solution. LPS was injected through the *vena jugularis externa* once at a dosage of 200 μg kg-1 BW on the 2nd day of the experiment (2-day-of-life). The LPS was administered with the use of syringe for insuline 1 cc sterile with needle G27, 0.40 × 13 mm U-40 in a total volume of 0.3 mL. Group LPS, was kept as a positive control received only *E. coli* LPS intravenously once at the dose of 200 μg kg −1 BW. Group K was kept as a negative control (K-) without any antibiotic treatment nor LPS administration. However, negative control received into *vena jugularis externa* an equal volume of *aqua pro injection*.

On the 6th and 14th day of chick’s life (6th and 14th day of experiment) the birds were euthanatized and spleen and thymus samples were subjected to the flow cytometric analysis.

### Flow cytometry

Fresh thymuses (from each chicks ten lobules; 5 from each side of the neck) and spleens were collected and single cell suspensions were immediately prepared. To examine the lymphocyte populations in thymus and spleen, single cell suspension were prepared by finely mincing the tissues and then pushing it through a 40 μm nylon cell strainer. Mononuclear leukocytes were isolated with density gradient centrifugation for 30 min at 720× g using Ficoll (1.077, Sigma Aldrich, Munich, Germany). The mononuclear leukocyte layer was collected, placed into separate tube, washed and centrifuged twice in PBS (pH = 7.4) at 720× g for 7 min at 4 °C. For immunophenotypic analysis, aliquots of spleen or thymus mononuclear lymphocytes (1x10^6^cells/ml) were incubated for 30 min with different mouse anti chicken monoclonal antibodies: RPE - CD3 (clone CT-3), FITC - CD4 (clone CT-4), RPE - CD8α (clone CT-8), FITC - Bu-1 (clone AV20), FITC - TCRγδ (clone TCR 1) obtained from Southern Biotech (Birmingham, AL, USA), and then washed three times with PBS (pH = 7.4). Spleen or thymus mononuclear lymphocytes were double stained with mouse anti-chicken FITC - CD4 and mouse anti-chicken RPE - CD8α, mouse anti-chicken RPE - CD3 and mouse anti chicken FITC-TCRγδ monoclonal antibodies. In addition, spleen mononuclear lymphocytes were single stained with Bu-1-FITC. Staining controls included cells incubated with appropriate isotype control antibodies (the mouse IgG 1-FITC and mouse IgG 1-RPE) (Southern Biotech, Birmingham, AL, USA) whereas unlabelled cells were used as a control for background fluorescence. For dual staining analyses, cell suspensions single stained with either FITC - CD4 or RPE - CD8α and RPE - CD3 or FITC-TCRγδ were also prepared to be used as compensation controls for flow cytometric analysis. Lymphocytes were then gated and the cells analyzed on FACSCalibur a flow cytometer (Becton-Dickinson Immunocytometry Systems, San Jose, California, USA). For each sample, 20,000 of gated lymphocytes were acquired, and the percentage of cell population was determined using CellQuest software (Becton Dickinson Immunocytometry Systems, San Jose, CA, USA).

### Statistical analysis

The significance of differences between results obtained was calculated using Student’s *t*-test (Statistica 9.0 software, StatSoft Polska Sp. z o.o., Kraków, Poland). In the first experiment the *t*-test was used to compare means of different groups (enrofloxacine, florfenicol, ceftiofur with control group) at a single time point. In the second experiment, first the means of the two groups (negative vs. positive control groups) were compared. Then, we compared means of E+LPS, F+LPS, C+LPS with positive control groups (LPS) using the *t*-test at a single time point. Significance was defined as *p* ≤ 0.05 or *p* ≤ 0.001. All data were expressed as the mean ± SD.

## Results

### Lymphocyte population in the thymus

- Experiment 1
  On the 6th day of experiment (d.e.), it was demonstrated decrease percentage of CD3^+^TCRγδ^−^ and CD8^+^CD4^+^ cells after enrofloxacine and florfenicol treatment in comparison to the control group (Table 1). On the 14th d.e. decreased percentage of CD3^+^TCRγδ^+^ cells after ceftiofur or enrofloxacine treatment, was shown (Table 1).

**Table 1:** The percentage of the cells in chicken thymus after antibiotics administration at 6th and 14th day of experiment (d.e)

The percentage of the cells in chicken thymus after antibiotics administration at 6 ^th^ and 14th day of experiment										
Group	CD3^+^ TCRγδ^−^		CD3^+^TCRγδ^+^		CD8^+^CD4^−^		CD8^+^CD4^+^		CD4^+^ CD8^−^	
	6th d.e.	14th d.e.	6th d.e	14th d.e.	6th d.e	14th d.e.	6th d.e	14th d.e.	6th d.e	14th d.e.
Control	68.46 ± 4.45	59.76 ± 3.85	6.74 ± 1.37	5.30 ± 1.08	6.27 ± 1.57	6.21 ± 1.57	79.35 ± 3.17	73.37 ± 3.82	5.69 ± 2.21	3.24 ± 0.78
C	65.13 ± 3.32	61.56 ± 3.37	5.43 ± 1.25	3.84 ± 1.35*	6.07 ± 1.40	6.81 ± 1.21	80.73 ± 2.10	75.32 ± 4.20	4.98 ± 0.79	3.11 ± 0.86
F	62.77 ± 3.08*	56.81 ± 1.88	5.66 ± 1.18	5.11 ± 1.71	8.67 ± 1.26*	7.89 ± 1.44	70.56 ± 2.15**	76.33 ± 4.47	8.00 ± 1.83	5.23 ± 1.21
E	58.73 ± 3.67**	62.61 ± 3.34	5.73 ± 1.51	3.53 ± 0.80*	6.90 ± 1.44	7.83 ± 1.73	70.64 ± 2.57**	75.27 ± 2.71	6.17 ± 0.57	6.10 ± 1.02

Chicks were housed in the cages (*n* = 4 cages), the experiment started on day 1st of chicks life (1st day of experiment, d.e). The group E (*n* = 14) was given enrofloxacin orally, the group F (*n* = 14) was given florfenicol applied to the crop each day during the 5-day treatment whereas the group C (*n* = 14) received in the first day of experiment single subcutaneous doses of ceftiofur sodium. The control group (*n* = 14) as a negative control was not treated by antibiotic. The measurements were taken on day 6th (*n* = 7 chicks per group) and 14th (*n* = 7 chicks per group). Thymus mononuclear lymphocytes were double stained with mouse anti-chicken FITC - CD4 and mouse anti-chicken RPE - CD8, mouse anti-chicken RPE - CD3 and mouse anti chicken FITC-TCRγδ monoclonal antibodies. Percentages of lymphocyte subpopulations within small thymus mononuclear cell population were analyzed on FACSCalibur flow cytometer. The significance of difference between the control and E, F, C groups was defined as * *p* ≤ 0.05 or **p ≤ 0.001; data were expressed as the mean ± SD
- Experiment 2
  In the group received *E. coli* LPS no significant differences were shown between positive and negative controls groups. However, an increased percentage of CD8^+^CD4^−^ and CD8^−^CD4^+^ cells was observed respectively in C+LPS and F+LPS or C+LPS and E+LPS groups in comparison to the positive control group (Table 2). No significant changes were shown on the 14th day of the experiment in the groups received *E. coli* LPS (data not shown).

**Table 2:** The percentage of the cells in thymus after antibiotics and LPS administration at 6th day of experiment (d.e)

The percentage of the cells in chicken thymus after antibiotics administration at 6th d. e.					
Group	CD3^+^ TCRγδ^−^	CD3^+^TCRγδ^+^	CD8^+^CD4^−^	CD8^+^CD4^+^	CD8^−^CD4^+^
	Mean ± SD	Mean ± SD	Mean ± SD	Mean ± SD	Mean ± SD
K(−)	72.06 ± 4.56	5.28 ± 1.82	3.04 ± 0.67	84.73 ± 4.10	2.35 ± 0.80
LPS	70.06 ± 4.08	5.15 ± 1.70	4.16 ± 1.28	80.74 ± 3.88	1.88 ± 0.26
C+LPS	61.70 ± 6.05	6.01 ± 2.05	6.64 ± 1.64**	80.43 ± 2.66	3.54 ± 1.09*
F+LPS	70.84 ± 2.70	3.90 ± 1.21	7.49 ± 2.13**	77.55 ± 4.56	2.30 ± 0.72
E+LPS	66.92 ± 3.69	4.08 ± 1.02	5.31 ± 1.31	84.42 ± 3.15	3.45 ± 0.71**

Chicks were housed in the cages (*n* = 5 cages of 14 chicks each), the experiment started on day 1st of chicks life (1st day of experiment, d.e). The group E+LPS (*n* = 14) was given enrofloxacin orally; the group F+LPS (*n* = 14) was given florfenicol during the 5-day treatment, whereas the group C+LPS (*n* = 14) received in the first day of experiment single subcutaneous doses of ceftiofur sodium. In addition, the chicks belonging to the groups: E+LPS, F+LPS, C+LPS received *E. coli* LPS. *E. coli* LPS was injected through the *vena jugularis externa* once at a dosage of 200 μg kg-1 BW on the 2nd day of the experiment (2-day-of-life). Group LPS, as a positive control received only *E. coli* LPS intravenously. Group K was kept as a negative control (K-) without any antibiotic treatment nor LPS administration. The measurements were taken on day 6th (*n* = 7 chicks per group) and 14th (data not shown). Thymus mononuclear lymphocytes were double stained with mouse anti-chicken FITC - CD4 and mouse anti-chicken RPE - CD8, mouse anti-chicken RPE - CD3 and mouse anti chicken FITC-TCRγδ monoclonal antibodies. Percentages of lymphocyte subpopulations within small thymus mononuclear cell population were analyzed on FACSCalibur flow cytometer. No significant differences were shown between positive (LPS) and negative (K-) controls groups. The significance of difference between the positive control (LPS) and E+LPS, F+LPS, C+LPS groups was defined as * *p* ≤ 0.05 or **p ≤ 0.001; data were expressed as the mean ± SD

### Lymphocyte population in the spleen

**Fig. 1:**
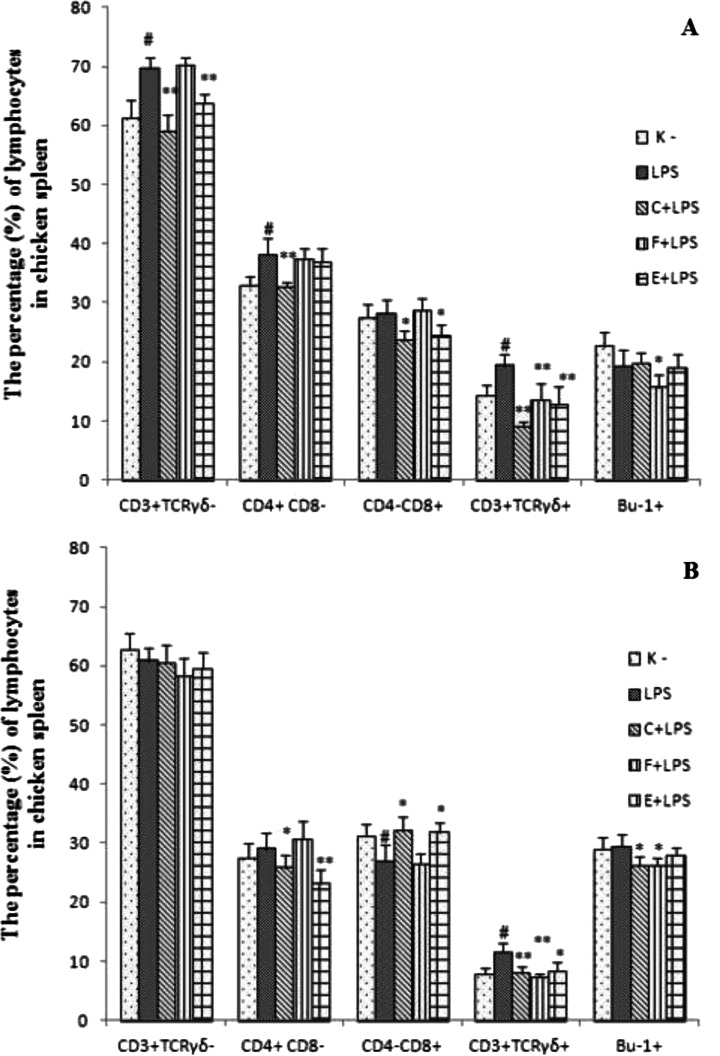
The percentage of lymphocytes in chicken spleen after antibiotics administration at 6th (**a**) and 14th day of experiment (**b**). Chicks were housed in the cages (*n* = 4 cages), the experiment started on day 1st of chicks life (1st day of experiment, d.e). The group E (*n* = 14) was given enrofloxacin orally, the group F (*n* = 14) was given florfenicol applied to the crop each day during the 5-day treatment whereas the group C (*n* = 14) received in the first day of experiment single subcutaneous doses of ceftiofur sodium. The control group (*n* = 14) as a negative control was not treated by antibiotic. The measurements were taken on day 6th (*n* = 7 chicks per group) and 14th (*n* = 7 chicks per group). Spleen mononuclear lymphocytes were double stained with mouse anti-chicken FITC - CD4 and mouse anti-chicken RPE - CD8, mouse anti-chicken RPE - CD3 and mouse anti chicken FITC-TCRγδ monoclonal antibodies and single stained with mouse anti chicken FITC - Bu-1monoclonal antibody. Percentages of lymphocyte subpopulations within small spleen mononuclear cell population were analyzed on FACSCalibur flow cytometer. The significance of difference between the control and E, F, C groups was defined as * *p* ≤ 0.05 or **p ≤ 0.001; data were expressed as the mean ± SD

**Fig. 2:**
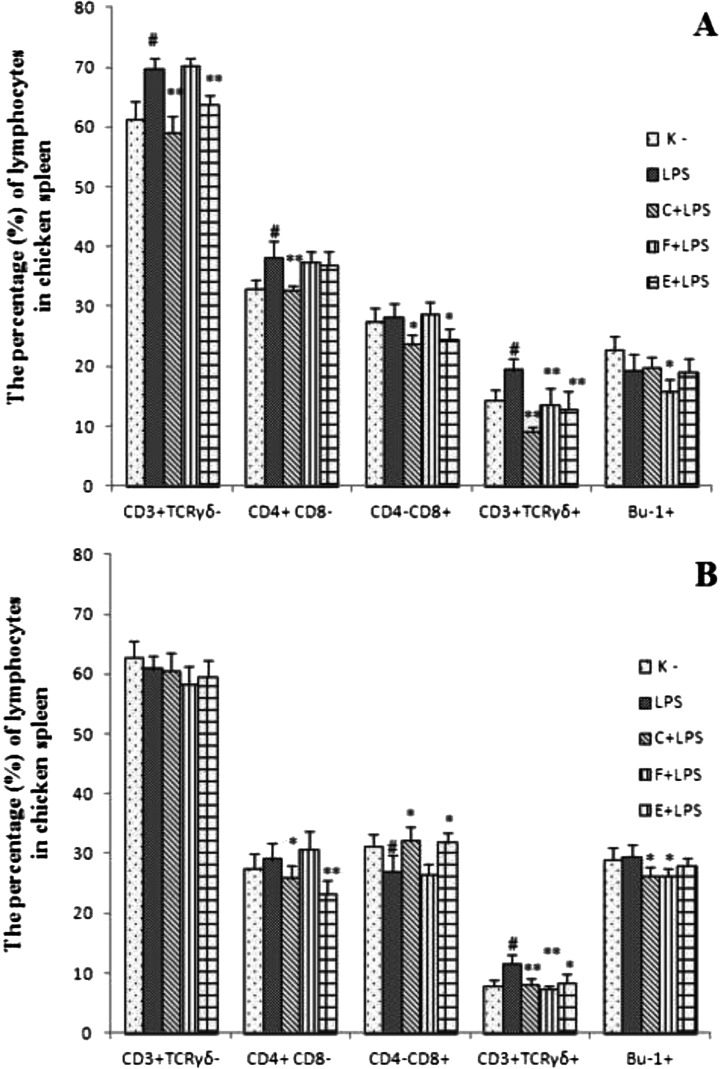
The percentage of lymphocytes in chicken spleen after antibiotics and LPS administration at 6th (**a**) and 14th day of experiment (**b**). Chicks were housed in the cages (*n* = 5 cages of 14 chicks each), the experiment started on day 1st of chicks life (1st day of experiment, d.e). The group E+LPS (*n* = 14) was given enrofloxacin orally; the group F+LPS (*n* = 14) was given florfenicol during the 5-day treatment, whereas the group C+LPS (*n* = 14) received in the first day of experiment single subcutaneous doses of ceftiofur sodium. In addition, the chicks belonging to the groups: E+LPS, F+LPS, C+LPS received *E. coli* LPS. *E. coli* LPS was injected through the *vena jugularis externa* once at a dosage of 200 μg kg-1 BW on the 2nd d. e. (2-day-of-life). Group LPS, as a positive control received only *E. coli* LPS intravenously. Group K was kept as a negative control (K-) without any antibiotic treatment nor LPS administration. The measurements were taken on day 6th (*n* = 7 chicks per group) and 14th (*n* = 7 chicks per group). Spleen mononuclear lymphocytes were double stained with mouse anti-chicken FITC - CD4 and mouse anti-chicken RPE - CD8, mouse anti-chicken RPE - CD3 and mouse anti chicken FITC-TCRγδ monoclonal antibodies and single stained with mouse anti chicken FITC - Bu-1monoclonal antibody. Percentages of lymphocyte subpopulations within small spleen mononuclear cell population were analyzed on FACSCalibur flow cytometer. The significance of difference between the negative (K-) and positive control (LPS) groups was defined as # *p* ≤ 0.05 whilst between the positive control (LPS) and E+LPS, F+LPS, C+LPS groups was defined as * *p* ≤ 0.05 or **p ≤ 0.001; data were expressed as the mean ± SD

The most significant changes were demonstrated in the spleen. The effect of antibiotics on a subset of T and B cells in the groups non receiving LPS was shown on Fig. 1 whereas the effect of antibiotics on a subset of T and B cells in the groups which received *E. coli* LPS was demonstrated on Fig. 2.

- Experiment 1
  In 6-days-old chicken, after florfenicol administration, a decreased percentage of CD3^+^TCRγδ^−^ and CD4^+^CD8^−^ cells was shown as compared to the control group. It was demonstrated, that after ceftiofur treatment the percentage of CD4^+^CD8^−^ was significantly lower while the percentage of CD4^−^CD8^+^ was significantly higher in comparison to the control group. A higher percentage of CD4^−^CD8^+^ cells was also shown after enrofloxacine administration. Additionally, a significantly lower percentage of Bu-1^+^ cells after ceftiofur or enrofloxacine treatment was demonstrated (Fig. 1a).
  On the 14th day of the experiment, it was shown decrease percentage of CD3^+^TCRγδ^−^, CD4^−^CD8^+^ cells after ceftiofur treatment. In addition, decreased percentage of CD3^+^TCRγδ^−^, CD4^+^CD8^−^ cells after enrofloxacine and florfenicol treatment. The percentage of Bu-1^+^ decreased significantly in all the experimental groups, as compared to the control group (Fig. 1b).
- Experiment 2
  The significant differences between negative (K-) and positive (LPS) control groups were observed in the second experiment. A higher percentage of CD3^+^TCRγδ^−^, CD3^+^TCRγδ^+^, CD4^+^CD8^−^ cells on the 6th day of the experiment and of CD3^+^TCRγδ^+^ on the 14th day of the experiment in LPS positive control group was shown as compared to the negative control (K-). A decreased percentage of CD4^−^CD8^+^ in the LPS group was shown on the 14th d.e. as compared to the negative control group.
  On the 6th d.e. the percentage of CD3^+^TCRγδ^−^, CD3^+^TCRγδ^+^, CD4^+^CD8^−^, CD4^−^CD8^+^ decreased in the C+LPS group as compared to the positive control group. Also, a lower percentage of CD3^+^TCRγδ^−^, CD4^−^CD8^+^ and CD3^+^TCRγδ^+^ in the E+LPS group was demonstrated in comparison to the positive LPS control group. A decreased percentage of CD3^+^TCRγδ^+^cells and Bu-1^+^ was found in the F+LPS group as compared to the LPS group (Fig. 2a).
  On the 14th d. e. a decreased percentage of CD4^+^CD8^−^ and increased percentage of CD4^−^CD8^+^ cells in the C+LPS and the E+LPS groups was shown as compared to the positive control group. In addition, decreased percentage of CD3^+^TCRγδ^+^ was found in all antibiotic and LPS received groups and Bu-1^+^ cells in the C+LPS and the F+LPS groups in comparison to the positive control group (Fig. 2b).

## Discussion

The enrofloxacin, florfenicol and ceftiofur have been widely used to treat a broad –array of infectious diseases. However, the effect of those antibiotics on the chicken immune system is still not clear. For instance, florfenicol may have a positive effect on cell mediated immune response and negative on humoral immune response (Khalifeh et al. 2009). In the other study it was found that florfenicol does not have any influence on immune response against NDV vaccine (Cao et al. 2004). In turn, Ellakany et al. (2007) reported that administration of 10 times overdosed enrofloxacin, significantly decreased the lymphocytic count in the peripheral blood. In our previously study, we have found that antibiotic treatment affects the number, percentage and distribution of B cells in the bursa of Fabricius in chicks (Chrząstek et al. 2011). To date, there is no data regarding the influence of antibiotic on chicken spleen and thymus cells. Here we have shown, for the first time, that enrofloxacin, florfenicol and ceftiofur administrated in therapeutic doses may decrease the percentage of B and T cells subset in lymphatic organs and therefore might influence on the proportion of B and T cells in chicken spleen and thymus. Moreover, it was demonstrated that these disturbances in proportion between the aquired immunity cells in thymus and spleen after antibiotics treatment were enhanced by *E. coli* LPS administration. Activation of the immune system by PAMPs, e.g., LPS is beneficial and helps the organism to recruit all its resources in order to fight the invading pathogen. This systemic response is characterized by high pro-inflammatory cytokine concentrations in the blood. However, anti-LPS agents have an ability to reduce the pro-inflammatory response by directly interacting with bacterial membrane and cell wall components making them unavailable to pattern recognition receptors. Here we demonstrated that enrofloxacin, florfenicol and ceftiofur have an capability to change the proportions among lymphocytes subset in lymphatic organs and thus might have an impact on the immune response to bacterial endotoxins in chicks. This action was especially manifested in splenocytes where we observed an opposite effect in the groups given antibiotics and *E. coli* LPS as compared to the LPS only treated group. While LPS contributed to the increase in the percentage of the cells, the antibiotics have decreased its. Recent studies done in mice have shown that those antibiotics can block NF-κB. Zhang et al. (2008) reported that florfenicol exerts anti-inflammatory activity. Similar result was found after ceftiofur or ciprofloxacine treatment (Purswani et al. 2002). Ci et al. (2008a) have shown that ceftiofur can inhibits TNF-α, IL-1β and IL-6 secretion in LPS-stimulated RAW264.7 cells (at 12 h post stimulation). Moreover, it was demonstrated that ceftiofur can also inhibit LPS-stimulated TNF - α secretion at 1 h post injection, IL-1 β at 12 h post injection and IL-6 at 3 h post injection in C57BL/6 mice (Ci et al. 2008b). In addition, Shuang et al. (2011) have demonstrated in mice that florfenicol not only suppressed Con A-, LPS- and OVA-induced splenocytes proliferation but also decreased the percentage of CD19^++^ B cells (B cells expressing the high levels of CD19) in a dose-dependent manner and suppressed CD3^++^ T cell (T cells with bright expression of CD3) at high doses. Similarly to mice splenocytes also in young chicks, we have found a decrease percentage of B (Bu-1^+^) and T cells (CD3^+^) after florfenicol treatment in therapeutic doses. In addition, the decreased percentage of those cells was also found after ceftiofur and enrofloxacine treatment.

The γδ T cells play an important role during bacterial infection (Schokker et al. 2012). Shibata et al. (2007) demonstrated that *E. coli*-induced IL-17 production (which is critical for the infiltration of neutrophils and bacterial clearance) by γδ T cells in mice, was triggered by TLR-4-mediated signaling. Here, we have shown that the percentage of those cells increased after *E. coli* LPS injection in the chicken spleen at 6th and 14th days of experiment. Thus, systemic injection of LPS may change the proportion of γδ chicken T cells in spleen what might be important during bacterial infection. In contrary, we found that systemic injection of LPS at the time of antibiotic treatment did not enhance the percentage of γδ T cells in the chicken spleen and therefore the percentage of these cells was similar to negative control. We can hypostatize that similar to mice cells, γδ chicken T cells respond to LPS stimulation, but antibiotics might have an capability to block TLR-4 signalling in chicken splenocytes. However, an additional study is needed to approve this suggestion.

To summarize, enrofloxacine, florfenicol, ceftiofur treatment may change the proportions among lymphocytes subset in lymphatic organs and thus might have an impact on the immune response to bacterial endotoxins in chicks. The antimicrobial agents that influence LPS-mediated immune response could further affect the cellular and humoral immunity that develops upon recovery.
